# Early Vessel Destabilization Mediated by Angiopoietin-2 and Subsequent Vessel Maturation via Angiopoietin-1 Induce Functional Neovasculature after Ischemia

**DOI:** 10.1371/journal.pone.0061831

**Published:** 2013-04-16

**Authors:** Di Qin, Teresa Trenkwalder, Seungmin Lee, Omary Chillo, Elisabeth Deindl, Christian Kupatt, Rabea Hinkel

**Affiliations:** 1 Medizinische Klinik und Poliklinik I, Klinikum Großhadern, Munich, Germany; 2 Department of Senile Disease, China-Japan Union Hospital of Jilin University, Changchun, Jilin, People's Republic of China; 3 Walter-Brendel-Centre of Experimental Medicine, LMU Munich, Munich, Germany; 4 DZHK (German Centre for Cardiovascular Research), partner site Munich Heart Alliance, Munich, Germany; Medical University Innsbruck, Austria

## Abstract

**Background:**

We assessed whether Angiopoietin-2 (Ang2), a Tie2 ligand and partial antagonist of Angiopoietin-1 (Ang1), is required for early vessel destabilization during postischemic angiogenesis, when combined with vascular growth factors.

**Methods:**

In vitro, matrigel co-cultures assessed endothelial-cell tube formation and pericyte recruitment after stimulation of VEGF-A, Apelin (APLN), Ang1 with or without Ang2. In a murine hindlimb ischemia model, adeno-associated virus (rAAV, 3×10^12^ virusparticles) transduction of VEGF-A, APLN and Ang1 with or without Ang2 (continuous or early expression d0-3) was performed intramuscularly (d-14). Femoral artery ligation was performed at d0, followed by laser doppler perfusion meassurements (LDI) 7 and 14. At d7 (early timepoint) and d14 (late timepoint), histological analysis of capillary/muscle fiber ratio (CMF-R, PECAM-1) and pericyte/capillary ratio (PC-R, NG2) was performed.

**Results:**

In vitro, VEGF-A, APLN and Ang1 induced ring formation, but only APLN and Ang1 recruited pericytes. Ang2 did not affect tube formation by APLN, but reduced pericyte recruitment after APLN or Ang1 overexpression. In vivo, rAAV.VEGF-A did not alter LDI-perfusion at d14, consistent with an impaired PC-R despite a rise in CMF-R. rAAV.APLN improved perfusion at d14, with or without continuous Ang2, increasing CMF-R and PC-R. rAAV.Ang1 improved perfusion at d14, when combined with rAAV.Ang2 (d0-3), accompanied by an increased CMF-R and PC-R.

**Conclusion:**

The combination of early vessel destabilization (Ang2 d0-3) and continuous Ang1 overexpression improves hindlimb perfusion, pointing to the importance of early vessel destabilization and subsequent vessel maturation for enhanced therapeutic neovascularization.

## Introduction

Molecular evidence demonstrates the feasibility of vessel growth in ischemic muscle tissue (for review cf. [1], [2]). If applied to patients suffering from manifest atherosclerosis of the heart or peripheral vessels, this fundamental principle would add to the therapeutic armamentarium of ischemic muscle disease. The surgical or interventional treatment options, though highly efficient and constantly improved, may at times be exhausted leaving a growing number of no option patients without functional revascularization.

Earlier trials with neovascularization therapy yielded inconsistent results [3] which indicated a necessity to further improve therapeutic agents or combinations thereof in order to translate the biological principle of balanced vascular growth into treatment. Currently, three distinct processes of neovascularization appear as minimal requirements for efficient vascular therapy: capillary growth (angiogenesis) and pericyte recruitment (maturation), followed by conductance vessel growth (arteriogenesis) [2]. In the recent years, microvessel maturation advanced to become an important target of therapeutic neovascularization, the lack of which would impair the efficient perfusion of microvessels and the successful growth of conductance vessels [4]–[6].

Physiologically, several processes mark the development of stable neovessels: first, degradation of extracellular matrix (ECM) and opening of pre-existing capillaries; second sprouting of endothelial cells forming a new tube consisting of tip cells at the top of stalk cells, third extension of endothelial tube guided into avascular area by gradients of angiogenic growth factors such as VEGF-A, fourth the assembly of new vascular basement membrane (BM) and finally recruitment of mural cells (pericytes and smooth muscle cells) to the neo-endothelial cells. Mature microvessels are required to efficiently perform blood perfusion providing nutrition and oxygen to the ischemic tissue. In contrast, immature capillaries lacking the investment of mural cell are prone to capillary regression and fading of an initial angiogenic response [7].

The Angiopoietin/Tie2 system regulates vascular maturation during development and postnatal angiogenesis. Ang1 binding to the Tie2 receptor has emerged as an essential event in the maintenance of a quiescent vasculature [8]. Ang1 acts by strengthening the contact between endothelial cells as well as endothelial cells and pericytes [9] and by increasing the migration of mural cells towards endothelial cells [10], [11]. Of note, Ang1 is also capable of induction of endothelial cell proliferation and migration [12], [13], and modulates VEGF-A induced neovascularization in vivo [14], [15]. Angiopoietin-2, in contrast, competes with Ang1 binding at the level of its receptor Tie2 inducing destabilization and regression of microvessels [16]. Ang2 transgenic mice reveal an impaired neovascularization after hindlimb ischemia induction [17].

For therapeutic neovascularization, a delicate balance appears desirable: whereas early destabilization may support the initial capillary proliferation process, subsequent maturation is required for the development of functional and lasting microvascular networks. In accordance, resistance to early destabilization may inhibit the sprouting of neovascularization and reduce the angiogenic response. Therefore, controlled early destabilization followed by enforced maturation of neo-vessels may constitute an attractive and novel concept of therapeutic angiogenesis in vivo. In the present study, we tested this hypothesis by evaluating Ang2 in combination therapies of three distinct profiles: In a first series, we assessed the combination of Ang2 and VEGF-A, a growth factor efficiently inducing capillary proliferation, but weakly providing capillary maturation [6], [7]. Second, we tested the combination of Ang2 and APLN, a growth factor providing both, capillary proliferation and maturation, leading to stable and non-leaky vessels [18]. Third, we assessed the potential of early Ang2-induced destabilization (d0-3) on top of continuous Ang1-overexpression providing neovessel maturation.

## Materials and Methods

### Animals

Animal care and all experimental procedures were performed in strict accordance to the German and NIH animal legislation guidelines and were approved by the Bavarian Animal Care and Use Committee (AZ 55.2-1-54-2531-130-8 and 35-12). All animal experiments were conducted at the Walter Brendel Centre of Experimental Medicine.

### Plasmids and virus

For cell transient transfection we used plasmid cDNA containing target gene (pcDNA, hVEGF-A, mAPLN, mAng2 and hAng1) controlled by the constitutive viral promoter cytomegalovirus (CMV). The tet-off gene expression system was utilized for mAng2 conditional turn-off by doxycycline (Dox) in the late stage. It contains two critical components: one is regulatory protein TetR and VP16. The latter converts the former to the hybrid protein tetracycline-controlled transactivator (tTA). Another is the response plasmid expressing the target gene with the minimal CMV promoter (PminCMV), and a tetracycline response element (TRE) upstream of the tTA which activates transcription in the absence of Dox. Production of recombinant adeno-associated viral vector was performed using a triple transfection method as described earlier [19]. In brief, plasmid one serves as a helper plasmid, plasmid two encodes for the AAV rep and cap sequences and the third plasmid encodes for the transgen including the CMV promoter. rAAV production is performed via U293 cell transfection and caesium gradient purification of the vectors [20]. Titer of the rAAVs was determined using rt-PCR utilizing the polyA tail of the vector bGH (primers: 5'-tctagttgccagccatctgttgt-3 forward, 5'-tgggagtggcaccttcca-3' reverse). Trans and helper plasmids were kindly provided by James M. Wilson, University of Pennsylvania. rAAV vectors were produced for the transgenes: CMV-LacZ, CMV-hVEGF-A, CMV-mAPLN, CMV-mAng2, CMV-tetoff-mAng2 and CMV-hAng1.

### Quantitative real-time PCR analysis

Successful transfections were confirmed by quantitative real-time PCR analysis (BIO-RAD, MyiQ, single color Real-Time PCR Detection System, Cat.No. 170-9740). The AAV vectors were applied to ischemic limb 14 days before artery ligation via i.m. injection. Muscle tissue of the lower limb (left and right gastrogenmius muscle) was obtained at day 7 after femoral artery ligation. qRT-PCR was performed as followed: Total RNA was extracted by using the Trizol Reagent-RNA/DNA/Protein Isolation Reagent (Molecular Research Center, Cat.No. TR-118) and transcribed into cDNA using IQ SYBR,RGREEN Supermix (BIO-RAD, Cat.No. 170-8882). Primers for detection of overexpression were: VEGF-A forward (5′- TTT TAC GCT ATG TGG ATA CGC-3′) and reverse (5′-AAG AGA CAG CAA CCA GGA TTT-3′); Ang2 forward (5′-TCG AAT ACG ATG ACT CGG TG -3′) and reverse (5′-GTT TGT CCC TAT TTC TAT C -3′); APLN forward (5′-GGC CAT CAC CAG CCA TTC CTT G-3′) and reverse (5′-GCA GCG TTA GCA GCA GCA TAG-3′); Ang1 forward (5′- GGT GGT TTG ATG CTT GTG G -3′) and reverse (5′- GGA TTC TAG TTG TGG TTT GTG-3′); S18 forward (5′-GACCCATTCGAACGTCGTCCCTATCAA-3′) and reverse (5′-GTAATTTGCGCGCTTGCTGCCTTCCTT-3′). The baseline and threshold were adjusted according to the manufacturer's instructions. The relative abundance of transcripts was normalized using the expression level of S18 mRNA. The data of the treated ischemic leg were compared to the sham operated leg and to non-treated control animals (Fig. S1).

### In vitro tube formation assay and co-culture

Human microvascular endothelial cells (HMECs) or murine endothelial cells (bEnd.3) were cultured in Dulbecco's modified Eagle's medium (DMEM +10% fetal calf serum (FCS) and 1% P/S); Pericytes (C3H/10T1/2, ATCC) were cultured in the complete growth medium including Eagle's Basal medium (BME) with 2 mM L-glutamine, 1.5 g/L sodium bicarbonate, Earle's BSS and 10% FCS. Expression of pericyte specific markers were analyzed via FACS measurements. After washing the pericytes in PBS, cells were incubated with primary antibody against NG-2 (Millipore AB 5320) and PDFG-R (abcam ab91066 rat monoclonal) for 45 min. Incubation with secondary antibody (for NG2 Molecular Probes anti-rabbit Alexa 488 A11008, for PDGF-R anti-rat sekundär: anti-rat-PE Santa Cruz sc-3740) was performed after washing of the cells for 45 min. Isotype controls were only incubated with the secondary antibody (Fig. S2A,B).

Tube formation of HMECs and co-culture of HMECs or bEnd.3 cells with pericytes in 2D matrigel assay was performed to analyse the interaction of pericytes and endothelial cells in vitro. ECs were transfected with pCMV-VEGF-A, pCMV-Ang2, and/or pCMV-APLN, pCMV-Ang1 in 24-well plate for 48 h. The co-transfection with two plasmids did not alter the expression level achieved via single pcDNA transduction. (Fig. S2) The ECs (HMECS and bEnd.3) were diluted to a final concentration of 1×10^4^ cells/50 µl in endothelial cell growth medium (ECGM)containing 5% FCS and 1% P/S, stained with DiD (red, Vybrant, Cat.No. 22887). Cells were plated on matrigel in angiogenesis μ-slides (ibidi, Cat.No. 81501). After 18 h tube formation was investigated and pictures were taken for quantification. The green stained pericytes (DiO, Vybrant, Cat.No. 22886) where added to the tubes in a concentration of 1×10^3^ cells/20 µl. After 6 h fluorescence pictures for red (endothelial cells) and green (pericytes) were taken and the pericyte per ring ratio was calculated.

### Mouse hindlimb ischemia model

In male C57BL/6J mice (n = 7 per group, Charles River, Sulzfeld, Germany) i.m. injection into the whole limb (10 injections 5 µl each) was performed with rAAV.hVEGF-A (high dose was 3×10^12^ virus particles, low dose was 5×10^11^ virus particles), rAAV.mAPLN, rAAV.hAng1, with or without co-application of rAAV.Ang2 or rAAV.tetoff-mAng2 (3×10^12^ virus particles), 14 days before surgery. Hindlimb ischemia model was performed as described previously. [21], [22] In brief, the right femoral artery was ligated distally of the profunda fermoral artery, whereas the ligature at the sham operated left femoral artery was only loosely winded around the artery at the same position.

### Laser Doppler imaging

Hind-limb perfusion was assessed using laser doppler perfusion imaging (MoorLDI-2; Moor Instruments, Devon, UK) [22]. After general anesthesia of the mice with 1.5% Isoflurane, mice were put in a heating chamber and maintained at 37°C. After an adaption time of 10 min in the heating chamber, measurements were conducted. LDI measurements were carried out before and after surgery on day 0 d, 7 d and 14 d. Results are given as right to left leg ratio including subtraction of background tissue value.

### Immunofluorescent histochemical staining

Tissue from the gastrocnemius muscle was harvested at day 7 (early timepoint) and day 14 (late timepoint) after femoral artery ligation. Tissue was embedded in tissuetech, stored at −80°C, cut to 5 µm slices and endothelial cells were stained with a CD31 goat anti-mouse polyclonal IgG (Santa Cruz, Cat.No. sc1506, dilution 1∶200) for primary antibody. A donkey anti-goat IgG-R (Santa Cruz, Cat.No. sc2094, dilution 1∶200) was used as secondary antibody. Pericyte staining was performed via NG2 staining (primary antibody of Chondroitin Sulfate Proteoglycan, Millipore, Cat.No. AB5320, dilution 1∶200) and secondary antibody of bovine anti-rabbit IgG FITC (Santa Cruz, Cat.No. sc2365, dilution 1∶200). Capillary density was analyzed as the ratio of CD31 positive cells per muscle fiber (CMF-R), and the maturation of capillaries was represented by pericyte to capillary ratio (PC-R). From each mouse 5 random pictures from the gastrocnemius muscle were analyzed.

### Statistical analysis

All data are presented as mean ± SEM and analyzed by SPSS 19.0 software. Comparisons among groups were made by one-way ANOVA testing with Gabriel's post hoc test. Games-Howell analysis was used for heterogeneity of variance. Differences between groups were considered significant for p-value of <0.05.

## Results

### Angiopoietin-2 antagonizes pericyte recruitment to growing tubes in vitro

**Figure 1:**
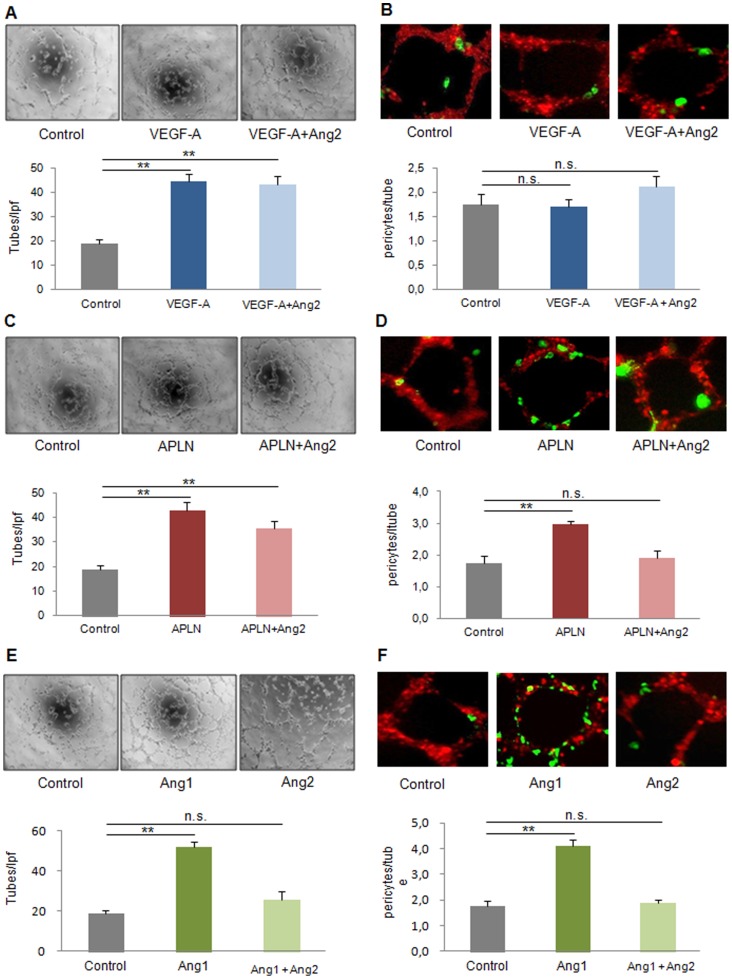
Capillary growth and maturation profile of VEGF-A, APLN and Ang1 with or without Ang2 in vitro. **(A)** Capillary-like tube formation was enhanced by VEGF-A alone transfection in HMECs (1×10^4^ per well of μ-slide angiogenesis plate) on 2D-matrigel. **(B)** 1×10^3^ Pericytes labeled by DiO (green) are plated after capillary-like tube formation of HMECs with DiD labeling (red). 24 h later, co-cultures reveal a low rate of pericytes attraction by VEGF-A, which was unaffected by Ang2. **(C)** Apelin (APLN) promoted tube formation with or without Ang2. **(D)** Pericyte recruitment was enhanced by Apelin, an effect attenuated by Ang2. **(E)** Ang1 induced tubes formationin the absence of Ang2. **(F)** However, the tube maturation provided by Ang1 was abolished in the presence of Ang2. (MEAN ± SEM, n = 5, ** p<0.01).

First, we sought to establish an *in vitro* model of pericyte recruitment to growing tubes. Therefore, DiD-labeled, growth-factor-transfected endothelial cells (HMECS or bEnd.3) embedded in matrigel were seeded with DiO-labeled pericytes. Tube analysis revealed that VEGF-A, despite robust tube formation (45±3 tubes/lpf vs. 19±2 tubes/lpf in control), did not alter pericyte recruitment (1.7±0.2 pericyte/tube vs. 1.8±0.2 pericytes/tube in control, Fig. 1A,B, Fig. S3A). In contrast, APLN, known to provide stable, non-leaky vessels in vivo [23], increased both, capillary proliferation (43±3 tubes/lpf, Fig. 1C) and pericyte investment (3.0±0.1 perictes/tube, Fig. 1D, Fig S3B). Ang1 was also capable of inducing tube formation and pericyte recruitment (Fig. 1E,F, Fig. S3C). Ang2, however, which did not alter the already limited pericyte attraction to VEGF-A treated endothelial cells, lowered the number of pericytes attracted to both, the APLN- (1.9±0.2 pericytes/tube) and the Ang1-transfected cell group (1.9±0.1 pericytes/tube). In vitro, tube formation capacity of APLN was unaffected by Ang2 (36±3 tubes/lpf, Fig. 1C), but suffered in the case of Ang1, when Ang2 was present (Fig 1E).

These in vitro experiments promped us to assess the effect of Ang2 in combination with VEGF-A, APLN and Ang1 in vivo.

### Early perfusion recovery of ischemic hindlimbs (d7) does not improve upon rAAV.VEGF-A, but decreases after combining VEGF-A+Angiopoietin-2

**Figure 2:**
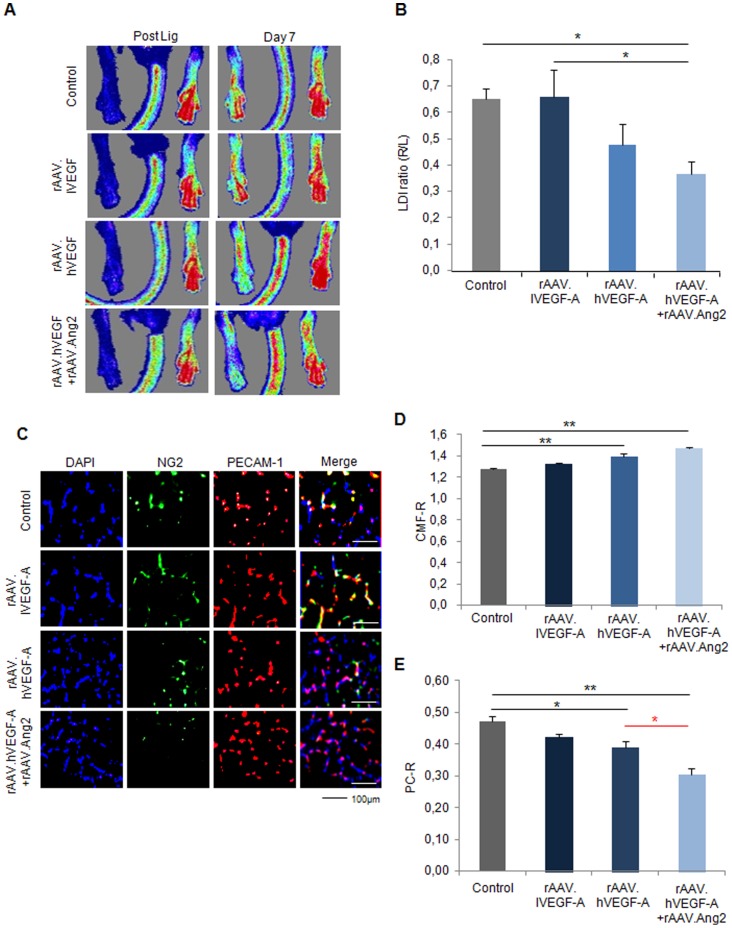
The failed therapeutic neovascularization after VEGF-A overexpression in the early stage (7 day) of mouse hindlimb ischemia model. (A) Laser Doppler Imaging (LDI) measured after right femoral artery ligation and at d7. (B) Quantification of LDI (ischemic versus non-ischemic hindlimb) revealed no significant neovascularization by VEGF-A at low (rAAV.lVEGF-A) or high doses (rAAV.hVEGF-A), but deterioration of perfusion in the rAAV.hVEGF-A+Ang2 group compared vehicle group (#P<0.05, vs. vehicle). (C) Fluorescence microscopy images of gastrocnemius muscle sections from control (rAAV.LacZ) or ischemic hind limb co-stained with DAPI (blue, nucleus), anti-NG2 (green, pericyte) antibody and anti-PECAM-1 (red, endothelial cell) antibody at d 7 after ligation. (D) Quantitative evaluation of capillary/muscle fibre ratio (CMF-R) demonstrated the ability of high VEGF-A with or without Ang2 to induce capillary growth in vivo. (E) Pericyte coverage was unaffected by lVEGF-A, but reduced in hVEGF-Agroup compared to vehicle group and even further reduced through the co-application of rAAV.Ang2. (MEAN ± SEM, n = 7,* p<0.05, ** p<0.01).

Employing a murine hindlimb ischemia model, we investigated the impact of VEGF-A, a well-characterized pro-angiogenic agent with weak vessel maturation potential. The overexpression of target genes (pCMV-hVEGF-A, pCMV-mAng2, pCMV-tetoff-mAng2, pCMV-mAPLN, pCMV-hAng1) as well as the reporter gene (CMV-LacZ), which were applied intramuscular via recombinant AAV (rAAV) vectors, was analyzed by qRT-PCR and histology (Fig. S1). Analysis of ischemic hindlimb perfusion by LDI indicated that rAAV.VEGF-A-either low dose (5×10^11^ virus particles, 0.66±0.10 LDI ratio (r/l)) or high dose (3×10^12^ virus particles, 0.48±0.08 LDI ratio (r/l))-did not alter the recovery of perfusion (0.65±0.04 LDI ratio (r/l) in control, Fig. 2A,B), whereas the combination of high rAAV.VEGF-A and rAAV.Ang2 significantly impaired perfusion recovery at d7 (0.36±0.05 LDI ratio (r/l)).

To explore the effect VEGF-A and Ang2 on angiogenesis capillary sprouting in vivo, we analyzed capillary/muscle fiber ratio (CMF-R, PECAM-1 staining) and pericyte/capillary ratio (PC-R, NG2 staining) by fluorescence histology (Fig. 2C-E). Of note, CMF-R was upregulated in the high rAAV.VEGF-A (1.40±0.03 CMF-R), but not in the low rAAV.VEGF-A (1.32±0.01 CMF-R) compared to control (1.28±0.01 CMF-R). In addition, the combination of high rAAV.VEGF-A with Ang2 revealed a significant higher CMF-R (1.47±0.01 CMF-R, Fig. 2D) than control group. In contrast, PC-R was decreased in the rAAV.VEGF-A/Ang2 group (0.31±0.02 PC-R) compared to control (0.47±0.02 PC-R) and low and high rAAV.VEGF-A treatment groups (0.42±0.01 PC-R for low and 0.39±0.02 PC-R for high VEGF-A, Fig 2E). Taken together, even though hVEGF alone and in combination with Ang2 significantly induces capillary growth, the failure to provide pericyte coverage is associated with a lack of perfusion recovery of the ischemic hindlimb, compared to controls and low VEGF. The combination of hVEGF and Ang2 even further decreased pericyte covarage and thereby showed a trend to a lower perfusion than the hVEGF alone.

### Late neovascularization (d14) is not provided by VEGF-A combined with Angiopoietin-2

**Figure 3:**
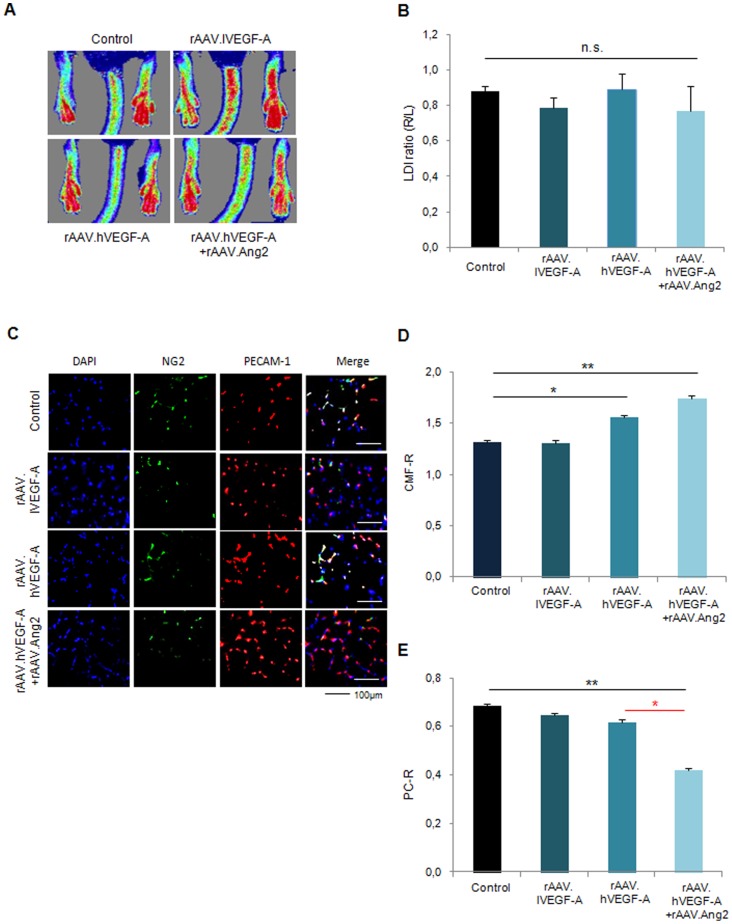
rAAV.VEGF-A did not provide enhanced neovascularization at d14. **(A, B)** Laser Doppler images and quantification of LDI of low and high VEGF-A groups displayed no difference to controls, similar to the combination of high VEGF-A with Ang2. **(C)** Fluorescence microscopy images of gastrocnemius muscle for capillaries (PECAM-1, red), pericytes (NG2, green) and nuclei (DAPI, blue) for the different groups. **(D)** Quantification of CMF-R displayed increased capillary growth for high VEGF-A with or without Ang2, whereas **(E)** PC-R revealed a diminished vessel maturation only for high VEGF-A combined with Ang2. (MEAN ± SEM, n = 7,* p<0.05, ** p<0.01).

In the next step we asked whether an intensified vessel destabilization allows for more capillary growth and a potential late gain of perfusion (d14). As indicated in Fig. 3A,B, whereas no significant gain of perfusion was achieved by rAAV.hVEGF-A only, the combination of VEGF-A and continuous Ang2 delivery, which impaired perfusion recovery at d7 (Fig. 2), did not impair perfusion at d14 compared to controls (0.88±0.03 LDI ratio (r/l) in control vs 0.77±0.14 LDI ratio (r/l) in hVEGF-A+Ang2). Histologically, the higher CMF-R in the hVEGF-A+Ang2 group (1.74±0.04 CMF-R vs. 1.31±0.02 CMF-R in controls, Fig. 3C,D) was offset by a significantly decreased PC-R (0.42±0.01), compared to control (0.69±0.02 PC-R) and VEGF-A muscle specimen (0.62±0.02 PC-R in hVEGF-A, Fig. 3C,E).

### Early Apelin-induced neovascularization is antagonized by permanent, but not temporary Angiopoietin-2 overexpression

**Figure 4:**
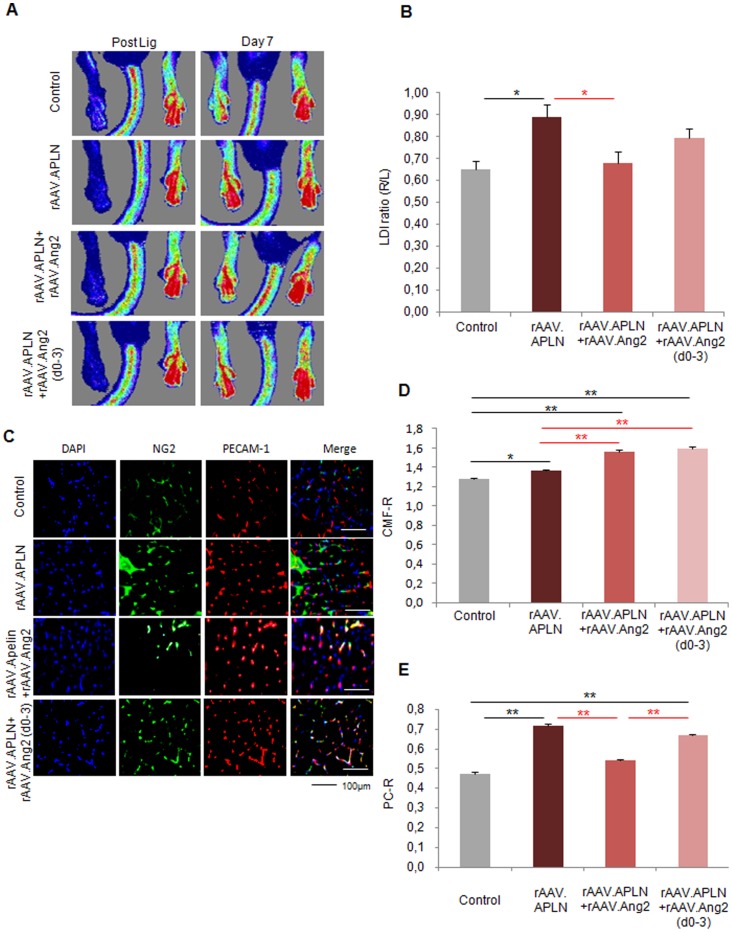
Apelin did not provide early neovascularization with Ang2. **(A, B)** LDI of blood reperfusion induced by APLN is enhanced without continuous Ang2 overexpression (rAAV.Ang2), where as the combination of APLN and Ang2 displayed no significant alteration. APLN combined with early Ang2 overexpression (rAAV.Apln+Ang2(d0-3)) induced a trend towards higher perfusion (p = 0.08). **(C–E)** PECAM-1 positive capillaries were slightly increased by rAAV.APLN, whereas the combination with continuous rAAV.Ang2 or rAAV.Ang2(d0-3) clearly enhaced capillary formation compared to APLN alone. Capillary maturation, indicated by pericyte coverage **(E)** was enhanced by rAAV.APLN with or without rAAV.Ang2(d0-3), whereas continous Ang2 reduced PC-R. (MEAN ± SEM, n = 7,* p<0.05, ** p<0.01).

Since VEGF-A did not provide a gain of perfusion in the ischemic hindlimb, we investigated the potential of apelin (APLN), a vascular growth factor inducing capillary sprouting and maturation, to improve perfusion after ischemia induction. Indeed, rAAV.APLN sufficed to significantly increase perfusion at d7 (0.89±0.06 LDI ratio (r/l), Fig. 4A, B). This effect was completely abolished by co-application of rAAV.Ang2 (0.68±0.05 LDI ratio (r/l)), in fact reducing the perfusion at d7 to control level. Histological analysis revealed that the gain of CMF-R induced by APLN (1.34±0.01) was even increased by rAAV.Ang2 coapplication (1.56±0.03) Fig. 4C,D). However, the PC-R, which was significantly increased upon apelin stimulation (0.72±0.01), was reduced to control level after rAAV.APLN/Ang2 co-application (0.54±0.01 PC-R, Fig. 4E). Thus, the improved early perfusion (d7) after APLN overexpression critically depended on pericyte recruitment. Preventing pericyte recruitment by Ang2-cotreatment sufficed to block a gain of perfusion upon apelin, despite an even improved capillary growth.

Next, we investigated whether temporary Angiopoietin-2 expression would improve the efficacy of APLN. Since we found that APLN induces vessel maturation in vitro, we compared both, either continuous or early vessel destabilization by Ang2. The latter was achieved by Doxycycline-inducible (Tet-off) Ang2, which allowed to switch off Ang2 expression significantly (Fig S1D). Using Doxycycline from d3 on, we limited Ang2 expression to d0-3 and investigated whether early Ang2 overexpression may boost APLN-induced vessel growth. Of note, we found a strong trend towards improvement of perfusion (0.80±0.04 LDI ratio (r/l), p = 0.08) (Fig. 4A,B) in the rAAV.APLN+Ang2 (d0-3) group, which co-incided with improved CMF-R (1.59±0.02) compared to rAAV.APLN only and improved PC-R (0.67±0.01), compared to rAAV.APLN+Ang2 (Fig. 4C–E). However, although early Ang2 overexpression favorably altered capillary growth and maturation indices at d7, it did not suffice to improve early hindlimb perfusion provided by APLN.

### Late neovascularization is provided by Apelin with or without Angiopoietin-2

**Figure 5:**
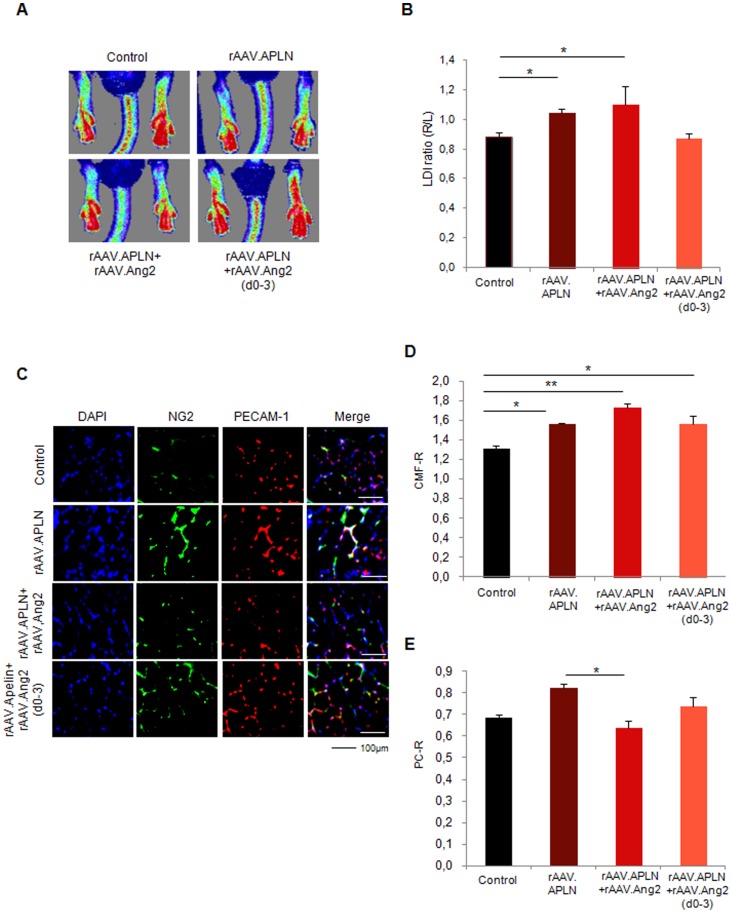
APLN provided late neovascularization, with or without continuous Ang2. **(A,B)** At d14, LDI indicated an increased perfusion by APLN with or without continuous Ang2 compared to control. Of note, the effect of APLN was prohibited by Ang2(d0-3). **(C,D)** At d14, rAAV.APLN improved capillary growth (CMF-R), when used alone or in combination with continuous Ang2 overexpression and temporary Ang2 (d0-3). **(C,E)** Pericyte recruitment was provided by APLN, but not if combined with continuous Ang2 overexpression. (MEAN ± SEM, n = 7,* p<0.05, ** p<0.01).

In contrast, rAAV.APLN provided a significantly increased perfusion at d14 (1.04±0.02 vs. 0.88±0.03 LDI ratio (r/l)), an effect still present during continuous co-expression of APLN and Ang2 (1.10±0.12 LDI ratio (r/l)), but not after APLN + Ang2 (d0-3) transduction, which provided Ang2 only in the first 3 days of postischemic vessel growth (Fig. 5A,B). The capillary/muscle fiber ratio increased in both, the rAAV.APLN and the rAAV.APLN+Ang2 groups and after rAAV.Apln+Ang2 (d0-3) transduction (Fig. 5C,D). The pericyte/capillary ratio, which was provided by APLN alone at d14, was significantly reduced in the APLN+Ang2-treated animals (Fig. 5C,E), whereas temporary overexpression of Ang2 showed a non-significant trend towards reduction of pericyte coverage. Thus, although APLN+Ang2 was efficient in improving capillarization at d14, a lack of mature vessels was associated with a lack of further improvement of perfusion beyond the level provided by APLN alone. When Ang2 overexpression is limited to the d0-3 period and switched off by Doxycycline application thereafter, Apelin fails to further increase perfusion above control level, and did not provide capillary growth or pericyte investment (Fig. 5A–E).

### rAAV.Angiopoietin1 requires temporary Angiopoietin2 expression for functional early vessel growth

**Figure 6:**
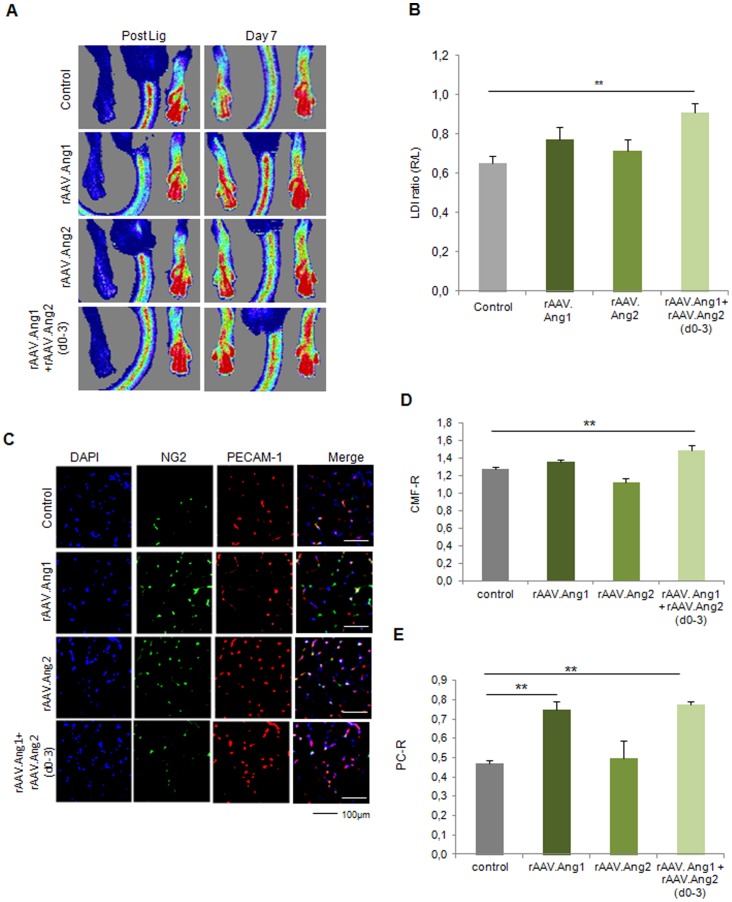
Ang1 combined with early Ang2 (d0-3) induces enhanced neovascularization at d7. **(A, B)** LDI analysis revealed no alteration of perfusion at d7 by either Ang1 or Ang2 (continuous overexpression), but a significant increase in the Ang1+Ang2(d0-3) group. **(C–D)** Analysis of capillaries revealed an elevated CMF-R only in the Ang1 and Ang2(d0-3) group, whereas the capillarization was unaltered if Ang 1 and Ang2 were overxpressed alone. **(C,E)** Ang1 alone as well as Ang1+Ang2(d0-3) was capable of enhancing pericytes coverage compared to control, Ang2 alone had no effect on the pericyte/capillary ratio (PC-R). (MEAN ± SEM, n = 7,* p<0.05, ** p<0.01).

Next, we assessed the potential of Angiopoietin-1, an antagonist of Ang2 and a powerful vessel maturation factor. Of note, neither Ang1 nor Ang2 induced significant increase of perfusion alone (0.78±0.06 LDI ratio rAAV.Ang1 and 0.72±0.06 LDI ration rAAV.Ang2, Fig. 6A,B). However, a combination of Ang1 and temporary overexpression of Ang2 (d0-3) (Fig. S1E) provided a significant gain of perfusion (Fig. 6A,B) at d7, along with a high capillary/muscle fiber ratio and a high pericyte/capillary ratio (Fig. 6C–E). rAAV.Ang1 and rAAV.Ang2 alone did not improve capillary growth (PECAM-1 positive cells, Fig. 6C,D), whereas pericyte coverage of the capillaries was enhanced after Ang1 overexpression and unaltered after Ang2 application (Fig. 6C,E).

### Late neovascularization is provided by Angiopoietin-1 combined with early Angiopoietin-2 treatment (d0-3)

**Figure 7:**
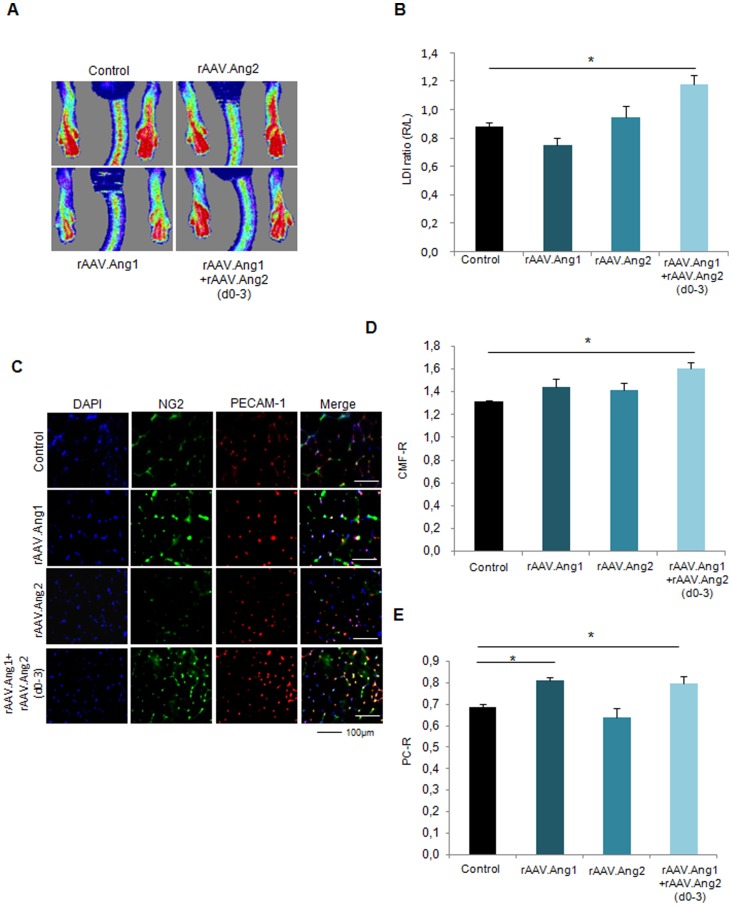
Ang1 combined with early Ang2 (d0-3) enhanced neovascularization at d14. **(A,B)** Ang1 improved hindlimb perfusion only, if combined with Ang2 (d0-3), a condition which also elevated **(C,D)** capillary/muscle fiber ratio (CMF-R) and **(C,E)** pericyte/capillary ratio (PC-R) to a significant higher level. (MEAN ± SEM, n = 7,* p<0.05, ** p<0.01).

Next, we assessed the late-timepoint efficacy of sequential Tie2-activation by Ang2 (d0-3) and continuous rAAV.Ang1 overexpression. In contrast to rAAV.Ang1 or rAAV.Ang2 alone, which failed to significantly exceed the control group´s perfusion at d14 (Fig. 7A,B), we could demonstrate a profound sustained gain of perfusion by combining Ang1 with early Ang2 overexpression (Fig. 7A,B). This effect was accompanied by an improved capillary/muscle fiber ratio (CMF-R) in the Ang1+Ang2 (d0-3) group (1.61±0.08 CMF-R, Fig. 7C,D) as well as an increase in pericyte coverage (0.80±0.03 PC-R, Fig. 7C,E). Neither rAAV.Ang1 nor rAAV.Ang2 provided increased CMF-R (1.31±0.02 CMF-R in control vs. 1.44±0.04 CMF-R (Ang1) or 1.41±0.13 CMF-R (Ang2)). Of note, whereas Ang1 increased PC-R, Ang2 decreased pericyte investment (0.64±0.04, Fig. 7C,E). Thus, an early vessel destabilization regimen, e.g. provided by Ang2 (d0-3), expanded the vascular growth response of rAAV.Ang1, which itself was capable of enhancing capillary maturation, but not capillary growth over time.

### Combination of Ang1 and Apelin does not provide enhanced neovascularization

**Figure 8:**
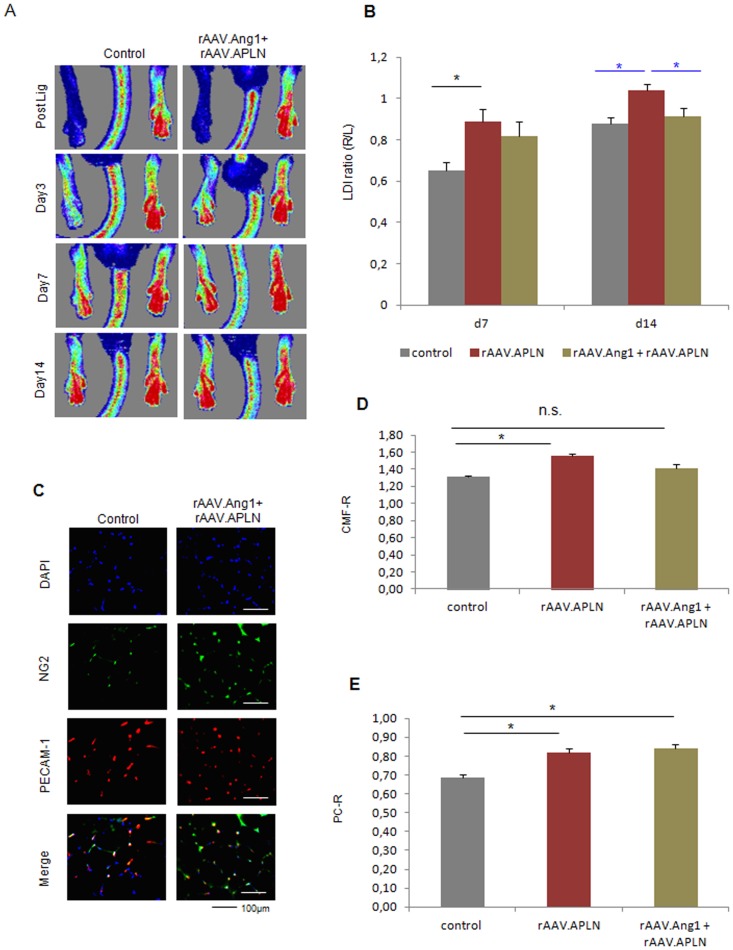
Apelin did not enhance neovascularization when combined with Ang1. **(A, B)** rAAV.APLN combined with rAAV. Ang1 was not capable of elevating hindlimb perfusion at d7 or d14 achieved by APLN alone. **(C–E)** The combination of APLN with Ang1 failed to increase capillary growth at d14, but provided a capillary maturation index (PR-R). (MEAN ± SEM, n = 7,* p<0.05, ** p<0.01).

Finally, we assessed tested the hypothesis that combination of two vessel maturation agents would not provide enhanced therapeutic vasularization. APLN, which itself provided increased perfusion of ischemic mouse hindlimbs (0.89±0.06 LDI ratio early and 1.04±0.02 LDI ratio late, Fig. 8A,B), was unable to do so when combined with Ang1, at the early (0.82±0.07 LDI ratio) or the late timepoint (0.91±0.04 LDI ratio, Fig. 8A,B). Moreover, Ang1 co-expression prohibited capillary growth provided (1.42±0.04 CMF-R) by APLN, but did not affect the pericyte recruitment induced by APLN alone (0.84±0.02 PC-R, Fig. 8C–E). These data indicate the possibility to prevent therapeutic neovascularization by capillary sealing with pericyte sheaths, which in fact decapacitated the otherwise effective growth factor APLN in combination with the second maturation factor, Ang1.

## Discussion

In this study, we elucidated the vital importance of the balance between capillary proliferation and stabilization in adult postischemic neovascularization. By virtue of continuously or temporarily active rAAV vectors, we assessed the role of early or continuous pericyte detachment via Angiopoietin-2. First, we found that VEGF-A failed to improve perfusion in a mouse hindlimb ischemia model, a finding further impaired by Ang2 a second vessel destabilization factor (Fig. 1, 2). Second, we found that APLN, itself a weaker capillary proliferating agent than high-VEGF-A, improved perfusion, associated with a high pericyte coverage (Fig. 1, 3). Addition of Ang2 to APLN, which enhanced capillary density in the ischemic hindlimb, delayed improvement of perfusion to the late time point (d14, Fig. 4,5). Combining APLN and early Ang2 (d0-3) did not improve perfusion in the same manner. (Fig. 4, 5)

Third, we observed that modulation of the Tie2 receptor by early Ang2 overexpression in addition to continuous Ang1 expression significantly increased hindlimb perfusion, associated with an increased capillary growth and maturation (Fig. 6, 7). Neither Ang1 nor Ang2 overexpression alone was able to accomplish this effect. Finally, the combination two vessel stabilizing agents (Ang1 and APLN) abolished therapeutic neovascularization achieved by APLN alone, but provided a very high maturation level, i.e. pericyte coverage (Fig. 8).

These results demonstrate that modulation of the delicate balance of vessel growth and maturation during postischemic neovascularization is a complex task. We first established that prevention of vessel maturation by adding Ang2 to VEGF-A-treatment is potentially harmful, most likely because VEGF-A itself is a powerful vessel destabilizing agent [5]. Here, further destabilization by Ang2 impaired early perfusion (d7), and no improvement above control levels at the later timepoint (d14), although VEGF-A+Ang2 treatment enhanced sprouting of vessels at early and late timepoints (Fig. 2, 5). These results fit the earlier observation in a genetic model of inducible endothelial Ang2 overexpression at a high dose, which suppressed recovery of neovascularization in the murine hindlimb ischemia model over 21 d [17]. Potentially, lower doses of Ang2 would be more desirable, since Felcht et al. recently observed a dual mode of action of Ang2 [24], acting pro-angiogenic on Tie2-low expressing endothelial cells, as opposed to the destabilizing activity of Ang2 on Tie2-expression endothelial cells [16]. On the other hand, it has repetitively been demonstrated that VEGF-A would act more efficiently in postnatal neovascularization, when coupled to a vessel stabilization agent such as Ang1 [15] or PDGF-B [6].

This strategy, a combination of a growth and maturation factor with a vessel destabilizing agent, was pursued by using either APLN or Ang1 in combination with Ang2. The neovasculatory potential of apelin was recently investigated, and a distinct antagonism to the VEGF-A induced hyperpermeability was revealed [23]. Of note, plasmid transfer of VEGF-A helped to raise the number of vessels induced by apelin, and provided an additional gain of perfusion compared to apelin alone. Consistently, in our study the limited capability of APLN to form new vessels, coupled with a high maturation potential (Fig. 4, 5), was improved by Ang2. However, this combination did not provide a further improvement of perfusion of the ischemic hindlimb, if used continuously, most likely due to the pericyte detachment induced by Ang2 (Fig. 5E). It is puzzling that the combination of APLN with early Ang2 overexpression did not further increase perfusion at the later timepoint (Fig. 5). Although the functions conceptualized before (early destabilization by Ang2 followed by enhanced maturation by APLN) can be demonstrated in vitro (Fig. 1) and in vivo (Fig. 3, 7), the efficacy of this approach is lacking in a clearcut and statistical significant manner. Obviously, this combination becomes dependent on the Ang2 effect, the subtraction of which by switching off the inducible vector compromises other components of the vessel growth process which cannot be compensated by APLN furtheron.

In order to rule out unforeseen effects of Ang2 on the maturation principle such as APLN, we speculated that combining early vessel destabilization via Ang2 with continuous Ang1 overexpression might provide a more balanced vessel growth than combination of Ang2 with other vasoactive agents. Since neither Ang1 nor Ang2 alone had an effect on perfusion recovery, we were surprised to note that the short Ang2-overexpression (d0-3), which did not increase the perfusion achieved in the APLN series (Fig. 4, 5), was highly efficient in raising the perfusion. Of note, several studies have reported a boost of the vascular growth response by late Ang1 addition [25]. Thus, remodeling the physiologic sequence of early Ang2 induction and subsequent Ang1 increase found in postischemic myocardial tissue [26] may serve the cause of early vessel destabilization and subsequent pericyte recruitment. The continuous Ang1 increase seems instrumental for vascular integrity and prevention of vessel regression, as observed in development [27] and in tumor vasculature [28].

The pro-angiogenic effects seen with the combination of a vessel destabilization (Ang2) and a subsequent vessel maturation (APLN or Ang1, Fig 5, 7) display a perfusion rate which is above 1, pointing to a supra-physiological perfusion. An improved perfusion index >1 after hindlimb ischemia in mice was already shown by Abe and colleagues. They demonstrated a blood flow recovery rate of 1.4–1.6 (LDI ratio R/L) at 14 days after repeated adrenomedullin pcDNA injection in a model of hindlimb ischemia. [29] In addition, values of LDI ratio right to left of 1 can already be achieved in control mice, depending on their age and genetical background. [30] [31] Furthermore, the operation technique (ligation vs. excision of the femoral artery) clearly influences the flow reduction and recovery capacity in this hindlimb ischemia model in mice. [30] [32] Taken together, these data confirm that a perfusion index >1 can be achieved, but might be dependent on the longterm or repeated overexpression of the right transgene. In addition, the mouse model of femoral artery ligation has high recovery capacity; therefore results should be viewed as a first evidence for a therapeutic treatment. Furthermore it allows some insights into signaling cascades and pathophysiological correlations. These concepts need to be reassessed in additional (more stable) animal models before translated to a clinical situation.

In summary, we showed that the concept of early vessel destabilization and late maturation is valid in providing enhanced postischemic hindlimb perfusion. We demonstrated that combining two destabilizing agents (VEGF-A and Ang2) at times deteriorates perfusion, whereas the combination of two growth and maturation factors (Ang1 and APLN) can even prohibit the efficacy of one (APLN) when applied alone. However, the choice of vascular agents is delicate: in our model, only the sequence of early Ang2 overexpression combined with continuous Ang1 overexpression provided enhanced perfusion, even superior to APLN, due to a induction of mature microvessels. However, this concept might add to the armamentarium of therapeutic approaches of chronic ischemic muscle disease.

## Supporting Information

Figure S1 **Efficacy of rAAV mediated growth factor transduction.** Intramuscular injection of rAAV.VEGF-A **(A)**, rAAV.Ang2 **(B)** and rAAV.APLN **(C)** into the ischemic limb displayed a significant increase of mRNA levels compared to the non-injected sham operated leg as well as to controls (displayed as ΔΔCT normalized to S18, day 7 post ligation). **(D)** Analysis of Ang1 levels revealed higher ΔΔCT levels at day 3 post ligation as well as on day 7, when Doxycycline was applied. **(E)** rAAV.Ang2 (day0-3) transduction showed enhanced Ang2 levels at day 3 (no Doxycycline) and a clear reduction of Ang2 at day 7 when the vector was shut off by Doxycycline. **(F)** I.m. injection of rAAV.LacZ revealed a clear blue staining for ß-Galaktosidase in the treated leg compared to control. (MEAN ± SEM, n = 3, ** p<0.05).
(TIF)

Figure S2 **In vitro expression analysis.** Pericytes (C3H/10T1/2) obtain from ATCC, express pericyte markers as NG2 **(A)** and PDGF-R **(B)** in FACS analysis. To asure that the co-transfection of two transgenes within one approach does not alter the expression level, RT-PCR analysis were performed. **(C,D)** APLN as well as Ang2 display the same expression level if transfected alone or in combination for ΔΔCT of APLN or Ang2. **(E;F)** Analysis of cells transfected with VEGF alon or in combination with Ang2 revieled similar expression levels for ΔΔCT of VEGF or Ang2 in both groups.
(TIF)

Figure S3 **In vitro pericyte recruitment.** **(A)** 1×10^3^ Pericytes labeled by DiO (green) are plated after capillary-like tube formation of bEnd.3 cells (murine endothelial cells) with DiD labeling (red). 24 h later, co-cultures reveal a low rate of pericytes attraction by VEGF-A, which was unaffected by Ang2. **(B)** Pericyte recruitment to the murine endothelial cells was enhanced by APLN, an effect attenuated by Ang2. **(C)** However, the tube maturation of the bEnd.3 cells provided by Ang1 was abolished in the presence of Ang2. (MEAN ± SEM, n = 5, ** p<0.01).
(TIF)
