# Supplementary figures and images for: Early Vessel Destabilization Mediated by Angiopoietin-2 and Subsequent Vessel Maturation via Angiopoietin-1 Induce Functional Neovasculature after Ischemia
Source: PLoS One. 2013 Apr 16;8(4):e61831. doi: 10.1371/journal.pone.0061831 (PMC3628915; doi:10.1371/journal.pone.0061831)

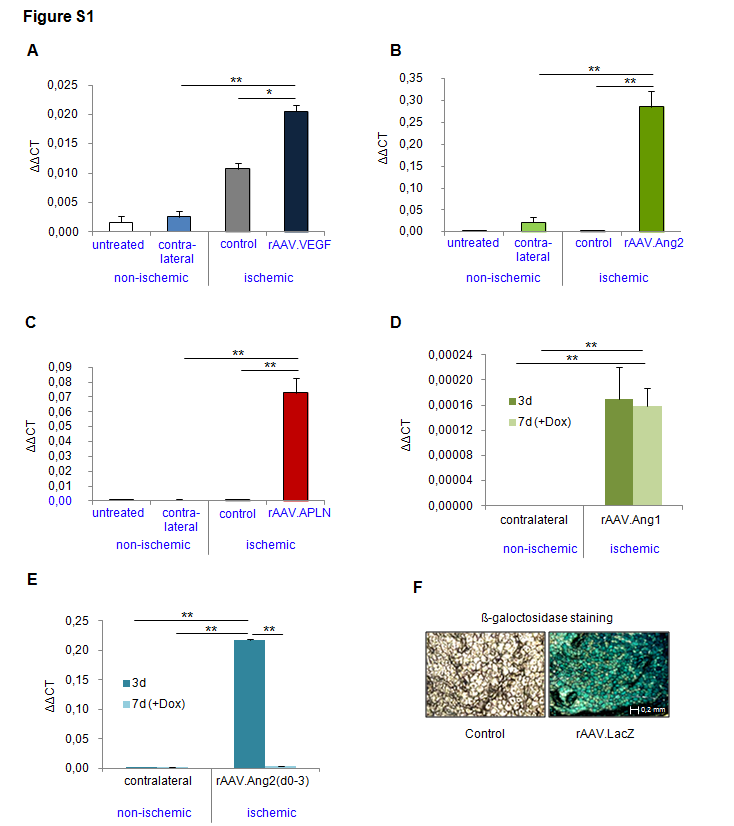

Supplement: Figure S1 — Efficacy of rAAV mediated growth factor transduction. Intramuscular injection of rAAV.VEGF-A (A), rAAV.Ang2 (B) and rAAV.APLN (C) into the ischemic limb displayed a significant increase of mRNA levels compared to the non-injected sham operated leg as well as to controls (displayed as ΔΔCT normalized to S18, day 7 post ligation). (D) Analysis of Ang1 levels revealed higher ΔΔCT levels at day 3 post ligation as well as on day 7, when Doxycycline was applied. (E) rAAV.Ang2 (day0-3) transduction showed enhanced Ang2 levels at day 3 (no Doxycycline) and a clear reduction of Ang2 at day 7 when the vector was shut off by Doxycycline. (F) I.m. injection of rAAV.LacZ revealed a clear blue staining for ß-Galaktosidase in the treated leg compared to control. (MEAN ± SEM, n = 3, ** p<0.05). (TIF) [file pone.0061831.s001.tif]

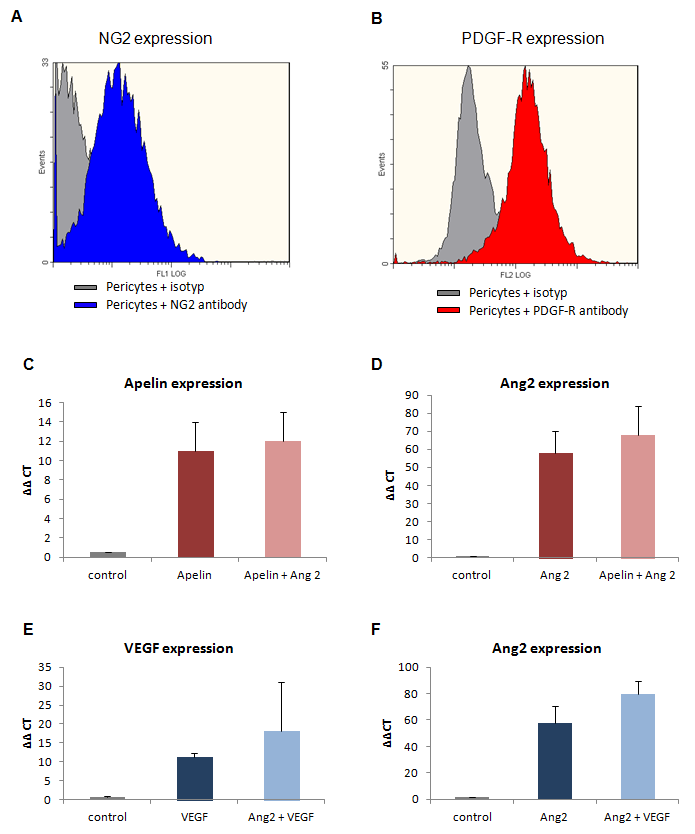

Supplement: Figure S2 — In vitro expression analysis. Pericytes (C3H/10T1/2) obtain from ATCC, express pericyte markers as NG2 (A) and PDGF-R (B) in FACS analysis. To asure that the co-transfection of two transgenes within one approach does not alter the expression level, RT-PCR analysis were performed. (C,D) APLN as well as Ang2 display the same expression level if transfected alone or in combination for ΔΔCT of APLN or Ang2. (E;F) Analysis of cells transfected with VEGF alon or in combination with Ang2 revieled similar expression levels for ΔΔCT of VEGF or Ang2 in both groups. (TIF) [file pone.0061831.s002.tif]

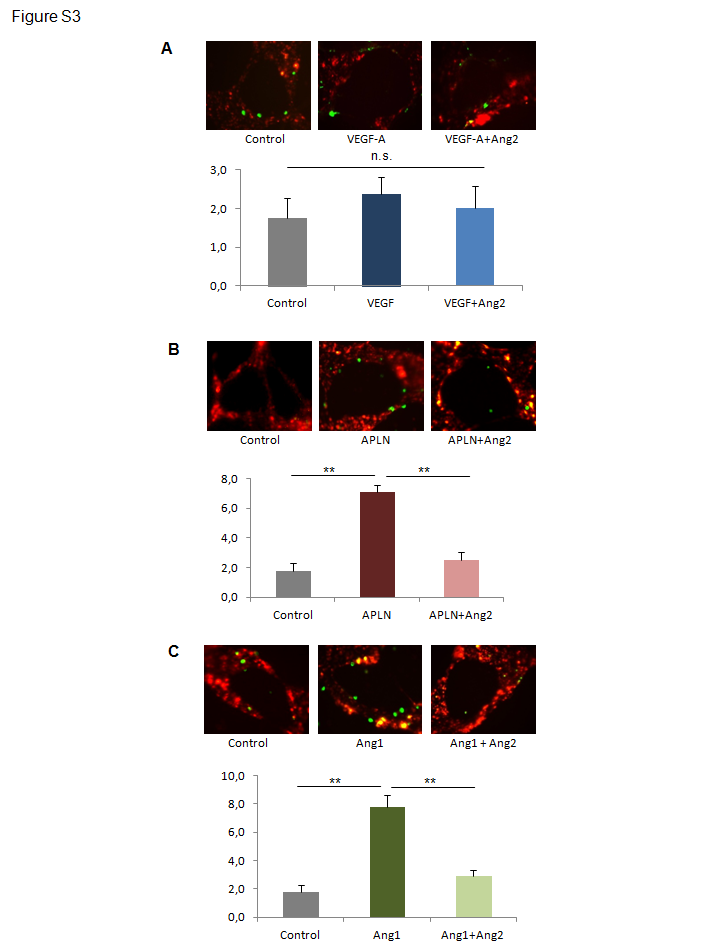

Supplement: Figure S3 — In vitro pericyte recruitment. (A) 1×10^3^ Pericytes labeled by DiO (green) are plated after capillary-like tube formation of bEnd.3 cells (murine endothelial cells) with DiD labeling (red). 24 h later, co-cultures reveal a low rate of pericytes attraction by VEGF-A, which was unaffected by Ang2. (B) Pericyte recruitment to the murine endothelial cells was enhanced by APLN, an effect attenuated by Ang2. (C) However, the tube maturation of the bEnd.3 cells provided by Ang1 was abolished in the presence of Ang2. (MEAN ± SEM, n = 5, ** p<0.01). (TIF) [file pone.0061831.s003.tif]
